# Choice of management strategy for papillary thyroid microcarcinoma: active surveillance or immediate surgery?

**DOI:** 10.7150/jca.91612

**Published:** 2024-01-01

**Authors:** Qi Liu, Mingyuan Song, Hao Zhang

**Affiliations:** Department of Thyroid Surgery, The First Hospital of China Medical University, Shenyang, China.

**Keywords:** Thyroid carcinoma, Papillary thyroid microcarcinoma, Overdiagnosis, Active surveillance, Outcome

## Abstract

Overdiagnosis of papillary thyroid microcarcinoma (PTMC) is prevalent, and effective management of PTMC is an important matter. The high incidence and low mortality rate of papillary thyroid carcinoma (PTC) justify the preference for active surveillance (AS) over immediate surgery (IS), particularly in cases of low-risk PTMC. Japan began AS in the 1990s as an alternative surgical option for PTMC and it has shown promising results. The safety and efficacy of AS management in PTMC have been verified. However, AS may not be suitable for all PTMC cases. How to find the balance between the decision-making of AS and IS requires careful consideration. Therefore, we collected and analyzed the relevant evidence on the clinical strategies for PTC and discussed AS and IS from the perspectives of health, economic, and psychological aspects, to help clinicians in choosing a more appropriate clinical strategy for PTC.

## Introduction

The incidence of thyroid carcinoma (TC) continues to increase worldwide due to the increasing number of papillary thyroid microcarcinoma (PTMC) diagnoses 1, 2. PTMC diagnosis is now more prevalent due to the rapid popularization of diagnostic imaging techniques, such as ultrasound and computed tomography (CT) 3, 4. Over 85% of TCs originate from follicular cells and are classified as papillary TC (PTC), which is considered a well-differentiated low-risk carcinoma. Only some rare subtypes, such as tall cell and columnar cell variants, are considered high-risk TCs 5.

Since 2010, the Korean Thyroid Association revised the guidelines for the diagnosis and management of thyroid nodules (TN) and TC 6, 7. These guidelines recommend fine needle aspiration cytology (FNAC) for > 10 mm nodules in patients with TC risk factors or cervical malignancy characteristics, which led to a significant increase in PTMC incidence in Korea. With this increasing incidence also came attention to the three “over” problems associated with PTMC: overdiagnosis, overtreatment, and over-staging. In studies of other cancers, breast cancer (BC) was found to be the most commonly overdiagnosed, followed by prostate cancer (PC), lung cancer, and TC 8. At present, multiple guidelines recommend a non-surgical strategy for PTMC, advocating active surveillance (AS) for low-risk PTMC instead of immediate surgery (IS) and converting to surgery only in case of tumor progression. In 2015, the American Thyroid Association (ATA) recommended that fine needle aspiration (FNA) not be conducted for small untouchable TN (< 1.0 cm) 9. However, contrary to the ATA guidelines, the Japan Association of Endocrine Surgeons recommends early fine needle puncture to promote staging and to guide clinical strategies 10. A prospective study in Canada showed that 71% of the patients with low-risk PTC (< 2 cm) preferred AS rather than IS 11. However, this was the initial preference of the patients, and the proportion of continuous AS may decrease with an increase in the follow-up time and change in psychological factors.

Much of the current controversy about overdiagnosis and overtreatment has focused on low-risk PTC because of a substantial reservoir of subclinical cancer and stable overall mortality 12. Although most low-risk PTCs are indolent, some of them show aggressive behavior, accompanied by lymph node metastasis 13, 14 and/or distant metastasis (0.5%) 15. In addition, PTMC can be sporadic or non-sporadic, with sporadic PTMC having a lower incidence of lymph node metastasis and a lower risk of recurrence than non-sporadic PTMC 16. Therefore, conservative treatment is recommended for patients with sporadic PTMC. Additionally, tumor location and other factors also affect the treatment strategy. For example, IS is recommended for PTMCs with potential recurrent laryngeal nerve or trachea involvement 17. In this review, we discuss the advantages and disadvantages of AS and IS for providing better clinical strategies for PTMC patients.

## The incidence of thyroid carcinoma has changed with the update of guidelines

At present, the incidence of several cancers, such as TC, BC, and PC, is on the rise, but mortality has not significantly increased. The improvement in diagnostic accuracy and reduction in mortality for BC and PC are associated with early diagnosis and intervention, suggesting that early clinical intervention is beneficial for these two cancers. Welch et al. proposed two explanations for the overdiagnosis of cancers: 1) that the suspected cancer did not progress or 2) that cancer progressed so slowly that there were no symptoms before the death of the patient besides for other reasons 18. However, the prerequisite for these explanations is that the tumor does not progress or that progression is not life-threatening.

In 1999, South Korea launched a National Thyroid Examination Project, which increased the incidence of TC in South Korea by 15-fold 19, and a similar phenomenon was observed in other countries 20. The incidence of TC began to decline after South Korea discontinued the Nationwide Thyroid Examination Project in 2014 21. Therefore, screening of all ages and populations is not appropriate, but it should be recommended in people with high-risk occupations and at the age of high incidence. A study by the National Cancer Institute of the United States (US) on TC prognosis revealed that the life table estimate of their 20-year cancer-specific survival rate of patients who received immediate treatment and those who did not receive immediate treatment were 99% and 97%, respectively 22, suggesting a similar prognosis for both strategies. This implies that although PTC progresses slowly, it may still pose a potential threat to life.

The incidence of TC continues to rise steadily in all high-income and developing countries, especially in China, Colombia, Lithuania, and Belarus, and especially in middle-aged women (35-64 years old) 23. One of the major reasons for the rapid rise of TC is that a large number of PTMCs are diagnosed. In China, the incidence of PTMC increased to 32.1% from 2000 to 2014 24. A study based on Surveillance, Epidemiology, and End Results (SEER) revealed that the incidence of PTC increased during 2000-2009 (APC 6.80, [95% confidence interval (CI) 6.46-7.13]), began to slow down during 2009-2014 (APC 2.58 [CI 1.71-3.47]), and declined annually since 2014 (APC -2.33 [CI 3.15 to -1.51]). In addition, distant metastasis of PTC decreased significantly from 2015 to 2018 (APC -17.86 [CI 26.47 to -8.25]). However, the mortality based on incidence increased during 2000-2018 (average APC 1.35 [CI 0.88-1.82]) 25, indicating that although the incidence declined after following clinical guidelines, the mortality based on incidence did not improve.

The European EUROCARE-2 study in 1985-1989 revealed that the 5-year overall survival (OS) of male and female TC patients was 72% and 80%, respectively 26. However, in this period, TC was not classified into different pathological types. Age-standardized relative survival (RS) rates for PTC during 1990-1994 in the EUROCARE-3 survey were 91% for men and 96% for women 27. Under the EUROCARE-4 study (2000-2002), Although classification by pathology was not performed, the age-adjusted 5-year survival rate for TC overall was 83.2%, compared with 93.5% for the contemporaneous US SEER-13 registries 28. The reduction in TC mortality in the US during the same period may have been a benefit from adherence to guidelines 29. The British summarized the reasons for the poor outcomes for TC compared to the European average to be due to incompliance the guidelines. When the UK began to promote clinical guidelines for TC, under the EUROCARE-5 study (2000-2007), the 5-year RS of PTC for men and women improved significantly, to 94% and 98%, respectively 30. The results of the EUROCARE-6-based study have not yet been published, and it is expected that more refined data on differentiated thyroid cancer (DTC), such as those on PTMCs, will be available for publication (Table 1). A study of PTMC in the US from 1995 showed that 16.7% of PTMC had recurrence 31. In a prognostic study of PTMC which began in 2000, also from the US, the recurrence rate after surgery was about 3%^32^. The data of these retrospective studies were collected before and after the publication of the ATA guidelines, and it may be inferred that the guidelines help improve the prognosis of PTMC. In addition to this, another National Cancer Database (NCDB) based study showed that patients with DTC who followed 2009 ATA or National Comprehensive Cancer Network (NCCN) guidelines had a significantly better 15-year DSS than those who did not follow the guidelines (78% vs 68%) 33. A retrospective study based on the SEER database showed that more patients with DTC received recommended surgical treatment after the publication of the 2006 ATA guideline, and the results indicated a significant improvement in 5-year DSS 34. The NCDB database and SEER database represent the majority of DTCs in the US, so these data may indicate that updated guidelines help improve DTC outcomes.

After a period of high incidence, due to the update of guidelines, the treatment strategy of TN or PTMC leaned towards non-surgical treatment 9, 35, which may be one of the reasons for the decrease in incidence in recent years. In the 1990s, the treatment effect of TC in the US was similar to that of BC today, and although the incidence of TC is rising and the mortality is declining 36, it does not imply that TC is inert. On the contrary, it is this decline in mortality that proves the effectiveness of normative treatment. The decline in mortality may not be the result of AS, but the outcome of early detection and early diagnosis.

## Comparison of advantages and disadvantages of AS and IS in PTC

AS was initially used in patients with localized PC and has been used in a variety of cancers, such as urethral cancer and intraocular melanoma 37, 38. These tumors are characterized by slow progress but with a risk of progression or metastasis. Sugitani et al recently published a survey emphasizing that > 50% of the low-risk PTMCs in Japan are under AS 39. At present, AS is not only limited to PTMC but also PTC with larger diameters, and even PTC with lymph node metastasis have been added to the AS cohort 40. A study showed that despite thyroidectomy, the cancer-related mortality of patients with PTC < 2 cm accounted for 12.3% of the total PTC deaths 41.

Following clinical guidelines, early diagnosis, and the participation of multidisciplinary management have decreased the mortality of most tumors. A retrospective study on AS found that there was no significant relationship between serum thyroid-stimulating hormone concentration and PTMC progression 42, 43. However, another study conducted in Kuma Hospital in Japan showed that levothyroxine treatment in patients is associated with decreased tumor growth during AS, but further studies are needed to confirm this result 44. Therefore, at present, there is no accurately monitored serum marker for AS follow-up in addition to ultrasound, and due to the lack of radiology or genetic indicators for PTMC progression, the risk criteria of PTMC cannot be determined at present with AS 45.

### Advantages of AS

Since the 1990s, Kuma Hospital in Japan has been using AS instead of IS for low-risk PTMC patients, and it has been implemented in other hospitals in Japan, the US, Korea, Italy, and China. Satisfactory results have been obtained after a long follow up. In a 30-year cohort study of AS and IS at Kuma Hospital in Japan, the 10-year and 20-year tumor growth rates were 4.7% and 6.6%, respectively. Only one patient in the AS subgroup developed distant metastases and none of the patients in this study died of TC 46. This long-term study confirmed the safety and feasibility of AS. In a prospective study of low-risk PTC, clinical outcomes were similar for AS and IS after a median of 37.5 months 47. More importantly, based on the current clinical research, TSH suppression therapy was shown to slow the growth of PTMC and improve the safety of AS 44, 48.

The significant reduction in surgical complications is one of the advantages of AS. Thyroidectomy may cause permanent hoarseness, permanent hypoparathyroidism, and iatrogenic hypothyroidism in 1%, 2%, and 4% of TC patients, respectively 9, 49, 50. Additionally, the permanent injury of postoperative hypocalcemia may be accompanied by serious symptoms or may even be life-threatening, requiring alternative treatment and long-term monitoring after discharge 51. IS may be associated with a higher rate of complications. However, there was also no increased risk of persistent or recurrent structural disease in AS compared to IS 47, 52. A recent study showed that there was no difference in surgical complications when AS was converted to surgery compared to IS 53. Based on the results of a large number of cohort studies on AS and IS, AS in small low-risk (primarily papillary) DTC is a relatively safe management strategy 54.

AS can avoid long-term TSH suppression treatment, and not only stabilize patients' emotions 55 but also reduce the risk of osteoporosis and cardiovascular disease 56, 57. In addition, AS is not only beneficial for the psychological and economic state of the patients, but it also helps avoid surgical complications, which can often lead to a decline in the quality of life (QoL). A cohort study of 222 patients with 4 years of follow-up found that patients in the IS group suffered significantly more anxiety 58.

Considering that the incidence and mortality increase with age, elderly people are susceptible to anesthesia-related complications, which carry a 0.5% incidence in patients over 80 years 59. Therefore, AS may be suitable for elderly patients as well as patients with multiple diseases who cannot tolerate surgery. Additionally, pregnant women are potential AS candidates, because thyroid hormones are critical for fetal development, and AS of low-risk PTMC patients can reduce the impact of thyroid hormone fluctuations on the fetus. However, if necessary, the best time for thyroidectomy is in the second trimester of the pregnancy 60. A study in Kuma Hospital showed that 8% of the low-risk PTMC patients showed a ≥ 3 mm increase in tumor diameter during pregnancy 61, and a retrospective study of 51 low-risk pregnant women with PTMC showed that 8% of the cases had tumor enlargement, but no cases had new lymph node metastasis 62. Although this may be attributed to hormonal fluctuations, further research is required to determine whether pregnancy is a risk factor for PTMC progression.

### Disadvantages and limitations of AS

The current version of the guidelines recommends AS for low-risk PTMC (T1aN0M0). However, the accuracy of the determination of low-risk PTMC remains unsatisfactory. A retrospective study of more than 900 PTMC cases showed that 9.6%, 5.6%, and 1.1% of the cases were in the T3, N1a, and N1b stages, respectively 63. Another retrospective study of 108 patients showed that Kuma criteria could not accurately predict the risk of PTMC. Among 29 patients with low-risk PTMC before surgery, 10 patients were confirmed to have clinical progression by postoperative pathology 64. Some PTMCs, although progressing slowly, can grow close to important blood vessels and nerves, and the enlargement of tumors can lead to vascular or nerve invasion. In DTC, vascular invasion is associated with tumor persistence/recurrence and short DSS 65. A Japanese research model showed that the possibility of lifelong disease progression in young TC patients is as high as 60% 66. Although multiple treatment modalities are now available for progressive thyroid cancer, a subset of resistant and progressive disease still can develop 67-69. Furthermore, some PTMCs, although small in size, can be highly invasive 16. Some pathological studies found that > 1 cm PTC may be associated with higher lymphatic vascular invasion (LVI) 70, although the impact of LVI on the prognosis of PTC is controversial. Some previous reports suggested that PTMCs of < 5 mm are usually not invasive, while those > 6 mm have a higher risk of lymph node metastasis 71. In a retrospective study, the sensitivity of correctly identifying lymph node metastasis in the central group was 22.6-55% 72. However, for micro-metastasis of lymph nodes, preoperative ultrasound sensitivity is also very low (26-56.2%) 73-75. Some PTMCs even showed skip metastases 76.

At present, molecular diagnosis of PTMC by FNA is still difficult, as gene mutations like BRAF cannot be identified. However, high-resolution ultrasound and ultrasound-guided FNA biopsy (FNAB) can be used to diagnose TN of ≥ 3 mm diameter 20, 77, 78. A SEER database study compared the biological behavior of the diffuse sclerosing variant, tall-cell variant, and classical PTMC and found that the former two subtypes were more invasive and had a greater probability of lymph node metastasis 79. This pathological type seems to be difficult to identify by cytology. Similarly, LVI cannot be detected by preoperative ultrasound and cytology. An analysis of the NCDB showed that the presence of LVI is independently associated with reduced survival in PTC patients 80, and since LVI is difficult to identify by AS, PTC patients with LVI may suffer from the risk of tumor progression or metastasis.

A 10-year AS study revealed that 7-16% of the patients required conversion to surgery, due to tumor growth (4-8%), cervical lymph node metastasis (1-2%), or the progression of other thyroid/parathyroid diseases or personal reasons (2-6%) 81. During the follow-up period, tumor enlargement or cervical lymph node metastasis was more likely to occur in young patients (< 40 years). An AS study in South Korea found that 14% of PTMCs can significantly increase in size, while 17% of TN can decrease in size during AS; however, these nodules were cystic and mostly benign 82. The change in tumor volume is determined by its biological properties. Although the current study showed a small difference in prognosis between AS and IS, there is currently insufficient evidence for the safety of AS.

Age is also a limitation of the AS selection strategy in PTMC. Compared to older PTMC patients, PTMC progression rate and tumor volume are higher in young PTMC patients 83, 84. In 2014, Ito et al. reported that the age of PTMC patients was an important factor in tumor enlargement and lymph node metastasis since the elderly group (> 60 years) were more likely to have tumor enlargement (p = 0.0014) and cervical lymph node metastasis (p < 0.0001) compared to the younger group (<40 years) 83. The elderly seem to be more suitable for AS, but paradoxically, it seems that the elderly are more prone to aggressive tumor subtypes 85. The recurrence rate of PTC in young people is higher, but the mortality is lower, while the elderly are more likely to show disease progression. However, a propensity score matching study showed that compared with the surgical group, the mortality of patients > 60 years would gradually increase with age 86. A study based on SEER data suggests that men ≥ 45 years of age or with PTC ≤ 2 cm should at least receive a lobectomy of the thyroid gland 41. With the update of guidelines, the age cutoff has been raised to 55 years old, but this does not reduce the risk of PTMC progression in elderly patients. Most of the PTMCs on AS are from Japan (Kuma Hospital in Kobe and Cancer Institute in Tokyo), while AS studies or the sample sizes in Europe and the US are insufficient. This racial difference will pose challenges in the global promotion of AS for PTMC.

### Current Indications and Suitable Candidates for AS

Patients with asymptomatic PTMC, without clinically significant lymph node metastasis, invasion of recurrent laryngeal nerve or trachea, high-level cytological manifestations, and distant metastasis are potential candidates for AS 87. However, it is difficult to effectively identify mucosal invasion of the trachea before surgery. Several studies have also demonstrated that AS is one of the viable alternative treatment strategies (Table 2). According to the recommendations of Memorial Sloan Kettering Cancer Center (USA), the ideal candidates for AS are older patients (> 60 years) with single focal PTMC and no evidence of lymph node metastasis 88; however, the AS strategy and the frequency of follow-ups for such patients were not discussed. As reflected in the latest version of the ATA guidelines, a lower-intensity treatment method can be adopted for low-risk TC, and AS can be used in appropriate patients to replace IS by observing the waiting and continuous neck ultrasound evaluation 7. AS is recommended for low-risk single-focus PTMC patients without thyroid extravasation and cervical lymph node involvement 20. However, the lack of a pathological diagnosis may lead to overlooking cervical lymph node micro-metastasis. At present, ultrasonic follow-up suggests that the following events be considered for PTMC patients under AS to switch to surgery: (1) the thyroid nodule increases by more than 3 mm compared with the initial value; (2) cytology confirmed metastatic lymph nodes in the neck; and (3) the excess of tumor volume increases by 30-50% 10, 89. At present, we tend to evaluate the safety of AS with the increase in tumor volume. Tuttle et al. conducted an AS study of 291 patients with PTC < 1.5 cm, and they observed tumor growth in 3.8% of the patients, with no local or remote metastasis during AS. However, it is worth noting that the median follow-up time was relatively short (25 months; 6-166 months) 89. This does not indicate the safety of AS for this slow-growing tumor. The clinical intervention rate for disease progression in 10 years is 8% after AS for TC, most of which is due to tumor volume increase 90. Whether patients with PTC are willing to coexist with the tumor is not only a psychological challenge but also a risk of tumor progression. The ATA guidelines define tumor enlargement as a 20% increase in the size of at least two nodules, with a minimum increase of 2 mm or a volume increase of more than 50% 9. Sugitani and Ito et al. believed that for low-risk PTMC patients, AS is a reliable alternative to avoid unnecessary surgery and surgical complications, while high-risk PTMC patients should undergo total thyroidectomy 10. According to the current Kuma protocol, PTC that met any of the following criteria was considered to belong to the high-risk group: tumor diameter > 4cm; tumor invasion of trachea or esophagus; diameter of metastatic lymph nodes > 3cm; distant metastasis. However, it may be difficult to distinguish low or high risk PTMC. NCCN suggest AS for low-risk PC patients with < 10 years of life expectancy, while it recommends either AS or surgical treatment for low-risk patients with >10 years of life expectancy 91. A cohort study from Kuma Hospital in Japan showed that young patients (< 40 years) with PTMC are more likely to experience tumor growth 48. Therefore, due to the lack of evidence, current guidelines do not recommend AS for children and adolescents younger than 20 years old 10. At present, many countries and organizations have updated the AS management strategy for DTC in their guidelines (Table 3).

In conclusion, AS is a safe one for PTMC and may replace one of the management options for surgical treatment strategies. But since there is still a lack of biological markers or imaging findings that reliably predict PTMC progression at present, more rigorous follow-up strategies and more appropriate indications for AS are needed.

## The psychological burden of AS and the choice of treatment strategy

### Terminology, a key factor in treatment decision-making

There are still some obstacles to the implementation of AS. Jensen et al. found that social beliefs about cancer, unclear surveillance protocols, and lack of supporting data are considered barriers to AS implementation in PTC 92. Terms such as cancer or carcinoma pose a great challenge to the psychology of patients, causing anxiety and panic. The moment when the doctors disclose the diagnosis and relevant information to the patients is crucial for patients to understand the severity of the disease. Additionally, the attitude of the doctors toward treatment also affects the patient's mood and decision-making strategies on treatment 93.

In 2016, a team classified the encapsulated follicular variant of PTC as a noninvasive follicular thyroid neoplasm with papillary-like nuclear features 94, thereby avoiding some sensitive terminology to reduce patients' anxiety. This reclassification or renaming resulted in approximately 45000 patients avoiding surgical treatment every year worldwide. In a randomized controlled trial, patients chose different treatment plans with different terminologies used for the diagnosis; approximately 19.6% of the patients chose total thyroidectomy when “PTC” was used, while only 10.5% and 10.9% of the patients chose total thyroidectomy when “papillary lesion” or “abnormal cells” were used, respectively 95. Therefore, emotions associated with disease terminology play a crucial role in treatment decisions. Fear and anxiety often cause patients to prefer thyroidectomy over AS. Additionally, thyroidectomy may give the patients as well as the surgeons a higher sense of security 96, as more than 33.4% of the doctors have been prosecuted for the delay in diagnosis 97. Among cancer patients, a major source of litigation is a delay in diagnosis, i.e., at an advanced stage of the disease, because clinicians do not schedule tests to detect cancer at an earlier stage. In TC, delay in diagnosis is the main cause of medical malpractice litigation in low-risk PTMC patients 98. However, clinicians may not readily accept these changes in methods and terminology of non-surgical treatment until new and stronger evidence emerges 99.

### Psychological burden of AS and IS

Most AS patients switch to surgical treatment due to persistent anxiety rather than tumor progression. However, the main concern of AS is that it may miss high-risk pathological types requiring radical surgery, which may cause a psychological burden. The psychological burden associated with AS and IS are summarized in Table 4. Patients with uncertain TN or PTC usually have a strong emotional response to their diagnosis, and their primary urge is to remove the tumor 96. Therefore, the new AS guidelines suggest eliminating the fear associated with cancer in PTC patients. Another clinical study of 200 patients showed that approximately 75% of the patients chose AS due to the fear of taking thyroid hormone post-surgery; however, upon disease progression during the follow-up, they preferred surgery 100. A study in the Netherlands showed that the health-related QoL (HRQoL) of AS and IS groups began to deteriorate over time; however, treatment with fluorodeoxyglucose positron emission tomography/CT (FDG-PET/CT) could help maintain a better HRQoL for one year 101. Although this may not be economical, it can reduce the anxiety of the patients.

A study revealed that the anxiety score of the AS group showed a downward trend, while that of the IS group was high for a prolonged period 102. However, anxiety seems to decrease in AS patients after a certain period of follow-up, such as 5 years 103. In contrast, Short-Form-12 or TC-QoL scores reveal that there is no difference in the QoL score between the AS and IS groups 104. Another study found that AS relies on better medical institutions and is related to anxiety and depression scale scores 105. In 2018, an 8-month follow-up study of the AS and IS groups in Korea showed that although the mental health status of the AS group was better than that of the surgical group (7.4 ± 1.3 vs 6.9 ± 1.6, p = 0.004), the group faced a greater fear of tumor progression or recurrence 106. Another AS QoL prospective study showed that PTMC patients receiving lobectomy treatment had more health complications than those receiving AS, mainly manifested as neuromuscular (p = 0.020), throat/mouth (p = 0.043), and scarring issues (p < 0.001) 104.

However, there are some opposing views. A State-Trait Anxiety Inventory study in Japan showed that the AS group had higher scores in anxiety (95% CI, -0.03-1.1; p=0.068) 107. A study in South Korea showed that after two years of AS, about 18% (101/561) of the patients withdrew from AS; among these, QoL did not decline for patients who switched to surgery due to disease progression, while the postoperative score declined for patients who switched to surgery due to anxiety or other thyroid diseases 108. However, another study revealed that treatment of TC, especially PTMC, with IS did not improve the psychological distress and sleep disturbance of patients compared with AS 109.

Therefore, based on the above evidence, we believe that to avoid psychological burden, it is important to eliminate the decline in QoL caused by psychological factors and to avoid long-term follow-up and constant anxiety caused by repeated ultrasonic examination. Thus, although ATA does not recommend FNA for TN < 1 cm, regardless of the ultrasound results, most people may opt for thyroidectomy for peace of mind 9, 90.

## Comparison of the economic advantages of AS and IS

The medical security of all the countries bears a heavy burden on the global economic slowdown. All the countries are looking for positive coping strategies for TC, and AS is an effective means to improve the burden of medical expenses. Although the medical systems of each country are different, the overall costs of AS and IS can be compared within the same medical system.

A study on the cost of AS and IS in Kuma Hospital showed that, in the absence of delayed surgery, the total cost of IS in 10 years was 4.7-5.6 times that of AS, while when switching from AS to surgery, IS was 4.1 times that of AS 110. Similarly, another study revealed that the total cost of IS in 10 years is 4.1 times that of AS 82. However, this is only a comparison of the total costs in 10 years, without considering the changes in long-term economic benefits. Different strategies should be adopted for PTMC patients based on the medical costs and healthcare systems in different countries. A study based on the Markov decision tree model shows that the incidental PTMC patients chose non-surgical treatment to save costs in 16 years compared with early surgical treatment, and after 17 years, although each patient spent more than USD 682.54, it gained an additional 0.260 quality-adjusted year 111. A similar Markov microsimulation model analysis shows that for small and well-differentiated PTCs, the ATA 2015 guidelines are highly cost-effective compared with the ATA 2009 guidelines, primarily because AS reduces the incidence of adverse events caused by surgery 112. Another study on 349 AS PTMC patients showed that the total cost of AS would increase after 16 years, compared with surgical treatment 113, eventually causing economic challenges for young AS patients. Another Markov decision tree model shows that surgical intervention is cost-effective in patients aged 40-69 years, and AS is more cost-effective than lobectomy for those > 69 years of age, with 17.3 quality-adjusted life years 114.

In addition to the comparison between IS and AS, the management of AS also has some economic research results. For instance, a 12-month RCT study found that close observation was more cost-effective than FNAC for patients with 1.0-2.0 cm TN 115. Another study showed that for 1.0 cm moderately suspicious TN, the cost of ultrasonic monitoring decreased by USD 1829 and increased by 0.016 quality-adjusted life years, compared with FNA 116.

## Conclusion and prospects

Several studies on low-risk PTMC patients confirmed the effectiveness and relative safety of AS, which was accepted by more patients and doctors. However, the indications and safety of AS in PTMC still need to be confirmed by large sample clinical studies. How to early detect PTMC with aggressive biological characteristics is one of the future concerns of AS. It is hoped that in the future, AI and machine learning will be used to judge the nature of tumors, and FNA will be used for molecular diagnosis to identify some tumors with rapid growth or metastasis potential, thereby improving the effectiveness and safety of AS. However, multi-disciplinary decisions are required to formulate the preferences and risk tolerance of different individuals to help patients choose between IS and AS for PTMC management.

## Figures and Tables

**Table 1:** Evolution of thyroid cancer treatment strategy based on EUROCAREs

Years	Research database	Region	Study Endpoints	Pathology	Results	Instructions	References
**1985-1989**	EUROCARE-2	European	1 year-OS 5 year-OS	All TC	Men 77% Women 83% Men 72% Women 80%	Pathological classification is not considered.	26
**2002**	EUROCARE-3	Britain United States	5 year-OS 5 year-OS	DTC	Men 64% Women 75% PTC 93%-94% FTC 84%-85%	UK begins customizing and implementing guidelines	27
**2000-2007**	EUROCARE-5	European	5 year-RS (00-07) 10 year-RS (05-07) 5 year-RS (00-07) in PTC	DTC	Men 81% Women 88% Men 79% Women 89% Men 94% Women 98%	The proportion of PTC continues to rise, while the RS also rises, indicating that standardized treatment is beginning to benefit.	30
**2009**	EUROCARE-4	Britain European	1 year-RS 5 year-RS 1 year-RS 5 year-RS	DTC	ALL 83.7% ALL 92.8% ALL 87.6% ALL 94.6%	The ratio difference of 5 year- RS/1 Year-RS was used as the diagnosis difference.	28

OS: Overall survival; RS: Recurrence survival; DSS: Disease free survival; TC: Thyroid carcinoma; DTC: Differentiated thyroid carcinoma; PTC: papillary thyroid carcinoma; FTC: Follicular thyroid carcinoma

**Table 2:** Clinical study and outcome of active surveillance in patients with papillary thyroid microcarcinoma

Author	Year	Cases	Follow-up (month)	Tumor size enlargement	Tumor volume increase	Development of lymph node metastasis	Reference
**Ito Y**	2014	1235	Mean, 60	4.6% in total 4.9%/5 years 8.0%/10 years	N/A	1.5% in total 1.7%/5-year 3.8%/10-year	83
**Fukuoka O**	2016	409	Mean, 81.6	6.0% in total 6.3% /5 years 7.3% /10 years	N/A	1.0%	117
**Tuttle RM**	2017	291	Median, 25	3.8% in total 2.5% /2 years 12.1% /10 years	12.4% in total 11.5%/2 years 24.8%/5 years	0%	89
**Kim**	2017	127	Median, 25	5.5%	20%	N/A	118
**Kwon**	2017	192	Median 30.1	2%	14%	0.5%	82
**Oh HS**	2018	370	Median, 32.5	3.5%	23.2%	1.4%	119
**Sanabria A**	2018	57	Median, 13.3	3.5%	N/A	N/A	120
**Sakai**	2019	392	Median, 88.8	15%	32%	3.8%	121
**Sanabria A**	2019	89	Median, 13.9	10% /2 years	23%	2.9%	122
**Molinaro E**	2020	93	Median, 19	2.1%	16%	1.1%	123

**Table 3:** Guidelines on indications for active surveillance (AS) and indications for conversion to surgery

Country or Organization	Year	Attitude towards AS	Follow-up management	References
**Japan Association of Endocrine Surgeons**	2018	Strongly recommended, high-quality evidence	Ultrasound evaluation is performed 1-2 times a year	124
**Chinese Association of Thyroid Oncology**	2016	Neutral attitude	Reevaluate every 6 months	125
**Korean Thyroid Association**	2016	Recommended	N/A	126
**American Thyroid Association**	2015	Strongly recommended, moderate-quality evidence	N/A	9
**Italy**	2018	recommended	Ultrasound evaluations should be performed every 6 months for the first 2 years and annually thereafter	127
**European Society for Medical Oncology**	2019	Strongly recommended	Every 6-12 months	128
**National Comprehensive Cancer Network**	2022	Recommended	N/A	129
**African Head and Neck Society**	2019	Recommended, high-quality evidence	N/A	130
**American Association of Endocrine Surgeons**	2020	Strongly recommended, moderate-quality evidence	AS requires specific patient counseling, selection, and a commitment to long-term follow-up.	131

**Table 4:** Results of the psychological evaluation of active surveillance (AS) and immediate surgery (IS)

Author	Years	Country	Subgroups	Research methods	Main Results	References
**Anna M Sawka**	2022	Canada	thyroidectomy vs. AS	Decision Self-Efficacy Scale	AS selection was independently associated with the fear of thyroid hormone supplementation post-thyroidectomy	100
**Rosa Falcone**	2020	Italy	advanced thyroid cancer (TC) vs. stable metastatic TC in AS vs. low-risk TC	Cancer—Quality of Life (QoL) questionnaire	Concerns about the impact of Covid-19 on follow-up and treatment	132
**Brooke Nickel**	2018	Australia	N/A	N/A	Terminology is an important factor in determining a patient's choice of treatment.	95
**Jae Hoon Moon**	2020	Korea	thyroidectomy vs. AS	QoL	QoL scores of anxious patients decreased after surgery, suggesting that personality itself affects QoL	108
**Allen S. Ho**	2022	USA	thyroidectomy vs. AS	18-Item Thyroid Cancer Modified Anxiety Scale	Anxiety scores in AS group showed a downward trend, while high anxiety scores persisted in the IS group	102
**Sung Hye Kong**	2019	Korea	AS v.s. IS	QoL	The mental health status of the AS group was better than that of the IS group; however, the fear of tumor progression was greater in the AS group compared to the IS group	106
**Min Ji Jeon**	2019	Korea	AS v.s. lobectomy (LB)	QoL	A higher level of health complications was observed in the LB group (neuromuscular, throat/mouth, and scar issues)	104
**Yusaku Yoshida**	2020	Japan	AS v.s. IS	State-Trait Anxiety Inventory	Patients in the AS group had higher anxiety scores	107
**Hiroko Kazusaka**	2022	Japan	AS v.s. IS	Short-Form 36 version 2)	Anxiety improves after a certain period of follow-up (such as 5 years) in the AS group	103
**Tomohiko Nakamura**	2020	Japan	AS v.s. IS	QoL	PTMC patients in the IS group had more complaints and high anxiety and depression	105
